# Structure and Dynamics of Adsorbed Dopamine on Solvated Carbon Nanotubes and in a CNT Groove

**DOI:** 10.3390/molecules27123768

**Published:** 2022-06-11

**Authors:** Qizhang Jia, B. Jill Venton, Kateri H. DuBay

**Affiliations:** Department of Chemistry, University of Virginia, Charlottesville, VA 22904, USA; qj3fe@virginia.edu (Q.J.); bjv2n@virginia.edu (B.J.V.)

**Keywords:** dopamine diffusion, fast scan cyclic voltammetry, carbon nanotubes, carbon microelectrodes, molecular dynamics, nanomaterials

## Abstract

Advanced carbon microelectrodes, including many carbon-nanotube (CNT)-based electrodes, are being developed for the in vivo detection of neurotransmitters such as dopamine (DA). Our prior simulations of DA and dopamine-*o*-quinone (DOQ) on pristine, flat graphene showed rapid surface diffusion for all adsorbed species, but it is not known how CNT surfaces affect dopamine adsorption and surface diffusivity. In this work, we use molecular dynamics simulations to investigate the adsorbed structures and surface diffusion dynamics of DA and DOQ on CNTs of varying curvature and helicity. In addition, we study DA dynamics in a groove between two aligned CNTs to model the spatial constraints at the junctions within CNT assemblies. We find that the adsorbate diffusion on a solvated CNT surface depends upon curvature. However, this effect cannot be attributed to changes in the surface energy roughness because the lateral distributions of the molecular adsorbates are similar across curvatures, diffusivities on zigzag and armchair CNTs are indistinguishable, and the curvature dependence disappears in the absence of solvent. Instead, adsorbate diffusivities correlate with the vertical placement of the adsorbate’s moieties, its tilt angle, its orientation along the CNT axis, and the number of waters in its first hydration shell, all of which will influence its effective hydrodynamic radius. Finally, DA diffuses into and remains in the groove between a pair of aligned and solvated CNTs, enhancing diffusivity along the CNT axis. These first studies of surface diffusion on a CNT electrode surface are important for understanding the changes in diffusion dynamics of dopamine on nanostructured carbon electrode surfaces.

## 1. Introduction

Rapid and precise in vivo electroanalytical detection methods have advanced through the development of carbon electrodes with novel micromorphologies, with the functional properties of these aqueous electrochemical interfaces depending on their surface structures [1,2]. In recent years, several carbon microelectrodes have been developed based on carbon nanotubes (CNTs), such as CNT nanoyarns [3], CNT forests [4], and spirally-wrapped CNTs [5]. CNT-based electrodes have been successfully employed to study dopamine (DA), an important neurotransmitter and signaling molecule in neuromodulatory processes [6,7,8] and a common target analyte for in vivo electroanalytical detection [2,9,10]. CNT-based electrodes have high sensitivity for neurotransmitter detection. CNT yarn electrodes have a limit of detection of 10 nM for dopamine and a linear range up to 25 uM [11]. Their response times are similar to those of carbon-fiber microelectrodes, and the electrochemistry is more reversible, meaning the reduction peak is more evident in the cyclic voltammetry.

Interestingly, scanning electrochemical microscopy (SECM) and scanning electrochemical cell microscopy (SECCM) experiments have shown substantial electrochemical activity on curved CNT surfaces. Pristine CNTs were able to catalytically oxidize and reduce ferrocenyl-methyl-trimethyl-ammonium [12], and the catalytic reduction of heavy transition-metal complexes were observed on pristine CNT sidewalls [13]. A pristine, curved CNT surface greatly enhanced the catalytic reduction of oxygen, as compared to a flat, highly ordered pyrolytic graphite (HOPG) surface [14].

At the same time, localized surface features, such as CNT kinks, oxidized defects, edges, and lattice defects, are all known to enhance electrochemical activity in these materials [2,14,15]. Spatially heterogeneous electrode activity has even been observed on pristine graphene surfaces [16]. Since analyte surface diffusion is much more rapid than adsorption and desorption [17,18], which occurs on the µs to s timescale for these small molecular analytes [19,20,21], the diffusion timescale of adsorbates between these functionally different locations has been used to explain certain features of electrochemical experiments, such as fast scan cyclic voltammetry (FSCV) scan-rate dependencies [22].

Surface curvature and roughness has been shown to influence adsorbate diffusion on a carbon surface. Shu et al. performed molecular dynamics (MD) simulations to study the surface diffusion of an adatom and found it highly dependent upon the CNT curvature and helicity, suggesting the possible importance of helicity in determining the mass transport of an adsorbate in the axial direction along a CNT [23]. In addition, an atomic force microscope (AFM) study on the clustering process of gold nanoparticles (NPs) on a single layer of graphene placed atop three different substrates—graphite, boronnitride, and SiO${}_{2}$—demonstrated that rougher carbon surfaces slowed NP diffusion [24].

Surface-dependent solvent dynamics can also play an important role in the diffusion of CNT-adsorbed analytes. Previous research has shown that water’s density, mobility, hydrogen-bond networks, and diffusion mechanism in close proximity to a CNT surface are highly dependent upon nanotube geometry [25,26,27]. The phase behavior and dynamics of water are also known to change under confinement, although these effects may be limited in spaces larger than 15 Å in diameter [27,28,29]. Interestingly, MD simulations have shown that the diffusion of water in certain regions within a CNT can be faster than in the bulk phase [25,30,31].

Our own prior work used atomistic MD simulations to investigate the diffusion dynamics of DA, its oxidation product, dopamine-*o*-quinone (DOQ), and their protonated species on the pristine basal plane of flat graphene [18]. The results demonstrated that the rapid adsorption of all species on this defect-free surface occurred even in the absence of a holding potential. In addition, we found that surface solvation has a large effect on adsorbate diffusion, and that adsorbate diffusivity on the solvated surface was similar to that in bulk water. Finally, we found that the protonated species diffused more slowly on the solvated surface, while the oxidized species diffused more rapidly.

In this paper, we extend our previous MD simulations of DA and DOQ on flat graphene to investigate the structures and diffusion dynamics of the same adsorbates on CNT surfaces of differing diameters, helicities, and arrangements. We first describe our model system and simulation techniques and then quantify the diffusivities of DA, DOQ, and their protonated species on various single CNT surfaces, at both the aqueous and vacuum interfaces. We then analyze how the relative populations of various configurations of adsorbed DA and DOQ change on differently curved CNTs, which provides insight into the origin of the observed curvature dependent diffusion. Finally, we discuss our findings regarding the surface diffusion dynamics of DA and DOQ in a CNT groove.

## 2. Modeling

Atomistic MD simulations were performed within LAMMPS [32] on systems composed of a graphene or CNT surface and a single adsorbate. Most simulations modeled the carbon:water interface, where the surface was solvated with TIP3P water molecules and simulations were conducted within an *NVT* ensemble. Simulations of the carbon:vacuum interface were conducted at fixed *NVE*. To model the interactions in the system, we used the OPLS-AA potential [33], which has been previously validated for use with small organic molecules on graphene surfaces [34,35]. Simulations followed the approach described in our previous study of DA and DOQ diffusion on a pristine flat graphene surface [18]. A detailed discussion of the accuracy of the OPLS-AA potential for these systems and additional details of the equilibration process can be found in that work [18] and the SI.

Although these surfaces operate as electrodes under an external voltage, the full analysis of adsorbate dynamics under a changing potential is remarkably complex and lies outside the scope of this work, which focuses instead on the fundamental influence of the graphene and CNT surfaces on the dynamics of adsorbed DA and DOQ.

### 2.1. Flat and Curved Pristine Carbon Surfaces

At the microscopic level, many carbon-based microelectrodes are composed of carbon fiber microfilaments and CNT yarns, which consist of disordered graphite and vertically-aligned CNT arrays, respectively [2,3]. To investigate adsorbate dynamics on the pristine carbon versions of these microelectrodes, we created periodic structures of flat graphene and single-walled CNTs of different curvatures and helicities. The surface carbons were immobilized during the simulations, as discussed in our prior work [18] and in keeping with other MD studies on these surfaces [23,26,27,30,31,34,36].

**Flat graphene.** We modeled a single layer of pristine flat graphene with a lateral box size of 98.2419×97.8420 Å${}^{2}$, using 3D periodic boundary conditions. A single layer of graphene was used as no differences were observed between adsorbate dynamics on a single and a triple layered fixed carbon surface [18].

**Figure 1:**
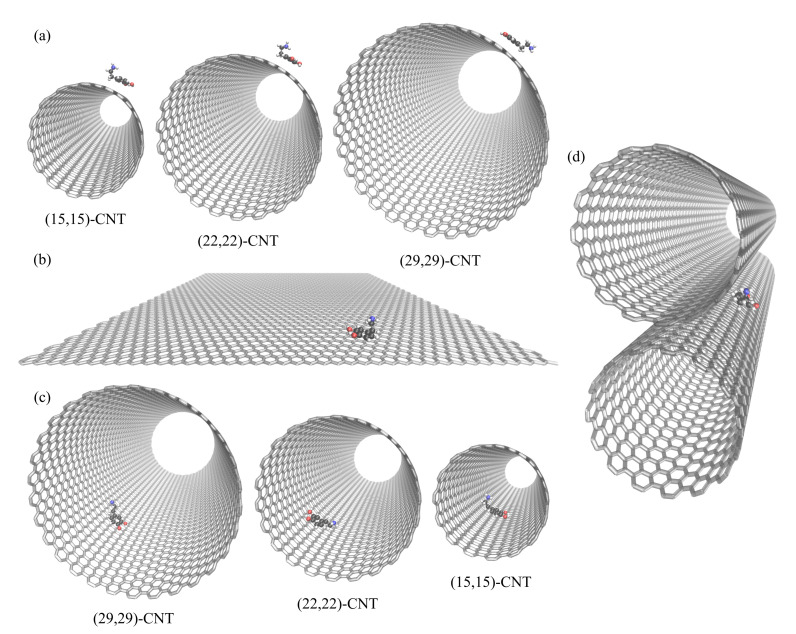
Simulated CNT and graphene surfaces. DA is shown on (**a**) the exterior surfaces of CNTs of varying curvatures, (**b**) flat graphene, and (**c**) the corresponding CNT interiors. In (**d**), DA diffuses along the exterior groove formed by two parallel (15,15)-CNT nanotubes. The dimensions of the CNTs are listed in Table S1, and the solvating water molecules were omitted here for visual clarity.

**Single-walled CNTs.** To look at analyte motion on various CNT surfaces, we modeled single-walled CNTs of three diameters and two helicities: armchair and zigzag. Armchair CNTs included (15,15)-CNT, (22,22)-CNT, and (29,29)-CNT, while the zigzag CNTs included (0,26)-CNT, (0,38)-CNT, (0,51)-CNT. Within each set, the radii are approximately 10, 15, and 20 Å, respectively. Details on the CNT diameters, lengths, and helicities are listed in Table S1. In addition, images of the CNTs can been seen in Figure 1. CNTs larger than 20 Å in diameter were chosen to avoid complications arising from significant confinement effects, which are more prominent in CNTs under 15 Å [26,27]. CNTs of of various lengths—ranging from 25 Å to 100 Å—were simulated in order to correct for finite size issues arising from the periodic boundary conditions, as discussed in the SI [18,37,38,39,40]. Results are generally presented from 100 Å-long CNTs; however, extrapolations to the infinitely sized systems are included for key cases.

**CNT groove.** We also placed two (15,15)-CNTs in a parallel alignment along the *z*-direction to construct a one-dimensional CNT groove. The CNTs are both 100.7 Å long and separated by 3.4 Å, corresponding to the sum of the van der Waals (vdW) radii of the closest carbon atoms on the different CNTs [41].

### 2.2. DA and DOQ Adsorbates

**Figure 2:**
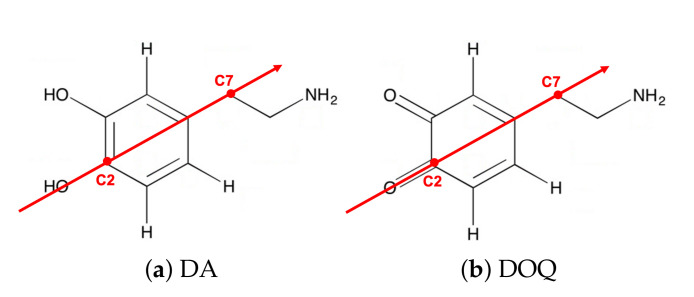
DA and DOQ. The adsorbate structures of DA and DOQ are shown here. The corresponding protonated species, DAH${}^{+}$ and DOQH${}^{+}$, have an additional hydrogen in their positively charged amine groups. The C2–C7 vectors (red arrows) are used to define the orientation and tilt of the adsorbates above the carbon surface.

We modeled DA and DOQ atomistically, along with their physiologically relevant protonated counterparts, DAH${}^{+}$ and DOQH${}^{+}$ [42]. A description of the partial charge assignments can be found in [18]. Cl${}^{-}$ ions were added as countercharges for the protonated species. DA and its derivatives contain three key moieties: the side-chain amine, the aromatic ring, and an *ortho*-diol or quinone group [43], see Figure 2. We also modeled the dynamics of a charge neutral atomic adsorbate with the same molar mass as dopamine (153.18 a.u.), which is referred to as “adatom(DA)”.

## 3. Results and Discussion

### 3.1. Solvated Adsorbate Diffusivities Depend on Surface Curvature

**Figure 3:**
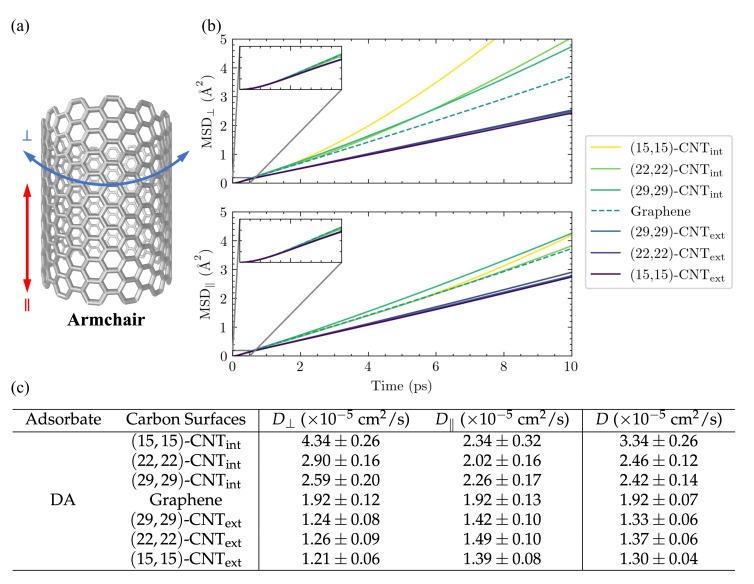
DA diffusion on differently curved carbon surfaces. Results are shown here for the diffusion of DA on the interior (int) and exterior (ext) surfaces of armchair CNTs of varying diameters and flat graphene. (**a**) The armchair designation [45] refers to the edge morphology of the CNT along the perpendicular direction. Diffusion on the surface in the same direction as the CNT axis is referred to as parallel (‖), while that around the circumference of the CNT is denoted as perpendicular (⊥). All CNTs presented in this table are 100.698 Å along the periodic ‖ direction, and the graphene sheet is 98.2419×97.8420 Å${}^{2}$ in size and periodic in two directions along the surface. (**b**) The MSDs as a function of time are shown in both surface directions: ⊥ (top panel) and ‖ (bottom panel). (**c**) The diffusion constants $D_{⊥}$, $D_{\|}$, and the overall 2D *D* values are computed from linearly fitting the MSD curves in (**b**) using the Einstein relation, Equation (S4), over the 4–10 ps range. In both (**a**,**b**), the carbon surface results are organized from most concave to the most convex.

Inspired by previous work showing that variations in surface curvature and CNT helicity can alter the diffusional pathways of an atomic adatom on a CNT surface [23], we set out to investigate the motion of DA across a series of solvated CNT surfaces with varying curvatures. The mean squared displacements (MSDs) of the adsorbates along the CNT axis (MSD${}_{\|}$) and around its circumference (MSD${}_{⊥}$) are plotted in Figure 3b as a function of time for DA on seven differently curved armchair CNT surfaces: three on the CNT interior, one on flat graphene, and three on the CNT exterior. The 1D diffusivities, $D_{⊥}$ and $D_{\|}$, and the overall 2D diffusivities, *D*, are listed in Figure 3c. The diffusion coefficients, *D*, were computed from the MSDs using the Einstein relation, [26,44] as detailed in the Supplementary Materials.

The observed diffusion constants are smallest on the convex CNT exterior and largest on the concave CNT interior. The largest shifts with curvature are seen in the $D_{⊥}$ values, in keeping with the direction in which the surface curves. In addition, a clear increase is seen in the $D_{⊥}$ values among the CNT interior results as the concavity increases from (29,29)-CNT${}_{\mathrm{int}}$ to (15,15)-CNT${}_{\mathrm{int}}$.

In order to compare the resulting diffusion constants to experimental values, we adjust them to correct for an unphysical system size dependence. This known finite-size effect [37,38,39,46] is discussed in more detail in the Supplementary Materials (see Figure S4 and accompanying text), and the values of the diffusion constants extrapolated to the infinite system size, $D_{\infty }$, are shown for a subset of the cases in Table 1. In our simulations, the extrapolated 2D diffusion coefficient of DA on flat graphene is $1.3\times 10^{-5}\;{\mathrm{cm}}^{2}/s$, while its value ranges from (1.1–2.5) $\times \;10^{-5}\;{\mathrm{cm}}^{2}/s$ for DA on differently curved CNTs. For comparison, the 3D diffusion coefficient calculated for DA from flow injection experiments is $0.6\times 10^{-5}\;{\mathrm{cm}}^{2}/s$ [47].

**Curvature dependence is observed for DA, DAH${}^{+}$, DOQ, and DOQH${}^{+}$.**Table 2 presents the diffusion constants obtained for these four species on both the interior and exterior surfaces of (15,15)-CNT, along with the flat graphene results. From these measurements, we find that the curvature-dependence is similar across all four species.

In addition, across all three curvatures we find that the protonated species, DAH${}^{+}$ and DOQH${}^{+}$, diffuse more slowly than their neutral counterparts, DAH and DOQ, while the oxidized species, DOQ and DOQH${}^{+}$, diffuse more rapidly than their reduced counterparts, DA and DAH${}^{+}$. These trends were previously observed on flat graphene [18] and can be readily explained by differences in the interactions of each species with the solvating water molecules: the positively charged species have increased interactions with the polar solvent, while the oxidized species have reduced interactions with the solvent—their quinone moieities are only able to act as hydrogen bond acceptors, as compared to the reduced diol moieties, which can act as both hydrogen bond donors and acceptors. Increased attractions with the solvent will increase the adsorbate’s effective hydrodynamic radius, $R_{H}$, which is inversely related to the diffusion constant, *D*, of a solvated sphere in nonturbulent flow via the Stokes–Einstein equation [36]: $D=\frac{k_{B}T}{c\pi \eta R_{H}}$, where $k_{B}$ is the Boltzmann constant, *T* is temperature, η is solvent viscosity, and *c* is a constant that describes the boundary conditions at the solvent-sphere interface. The Stokes–Einstein equation cannot be rigorously applied here for these partially solvated, small molecular adsorbates; however, it qualitatively explains the observed trends.

Our simulation results show that DA diffusion clearly depends on placement on the inner or outer surface of the CNT, with enhanced motion on the CNT interior. Overall, this observed curvature dependence is consistent with the general trends observed previously for an atomic adatom [23].

### 3.2. Dependence Does Not Arise from Curvature-Induced Shifts in Surface Roughness

In the case of the previously studied atomic adatom, the observed reduction of diffusion barriers for the adatom on a surface with negative curvature (the CNT interior) resulted from the smoothing of the carbon energy surface as it changes from convex to flat to concave [23]. However, it is not clear how this effect functions for a molecular adsorbate such as DA, which is larger than the underlying hexagonal carbon structure, flexible, and asymmetric in shape with an uneven charge distribution. In addition, the role of solvent was not considered in the prior work and may mitigate the influence of surface energy roughness. In this section, we probe the role of surface roughness in this system by investigating how the lateral distributions of these adsorbates depend on curvature, how their diffusivities depend on CNT helicity, and how their diffusivities depend on curvature in the absence of solvent.

**Figure 4:**
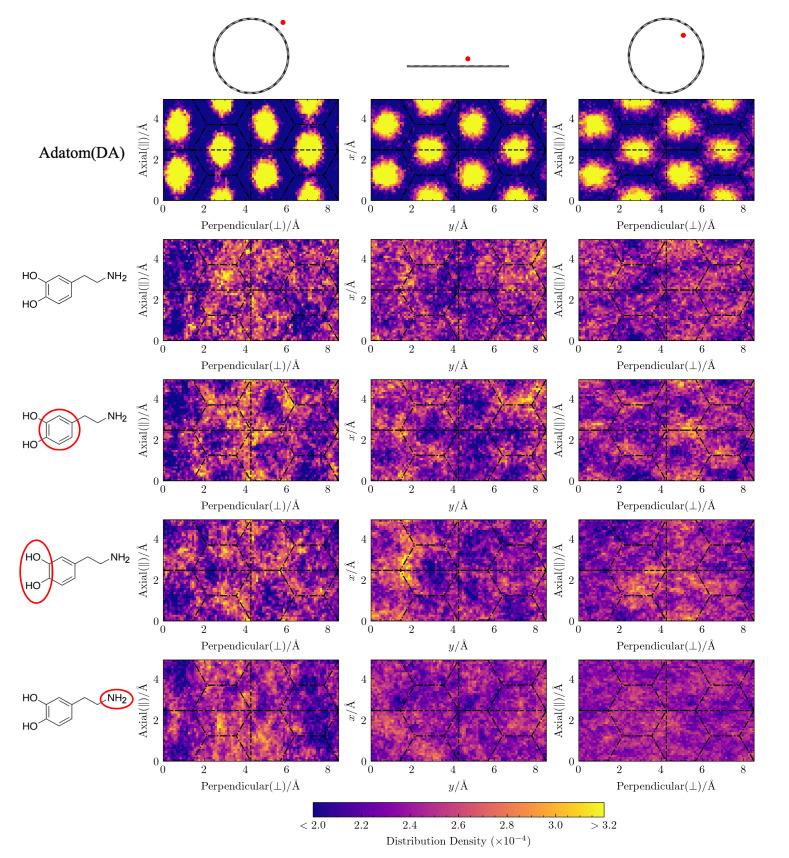
Lateral distributions of adatom(DA) and DA above the CNT and graphene surfaces. The plots show the distribution densities of the adsorbates above the carbon surface. From left to right, the columns show the distributions on the exterior surface of (15,15)-CNT, on flat graphene, and on the interior surface of (15,15)-CNT, as indicated with the cartoon images above each column. From top to bottom, the rows show the results for adatom(DA) (an atomic adatom with the mass of DA), the COM of DA, and the three COMs of the red-circled DA moieties. The projected COM coordinates are binned with a spatial resolution of 0.1×0.1 Å${}^{2}$ and wrapped into 4 unit cells, which are separated by the dashed lines.

**Lateral distributions of molecular adsorbates are similar across curvatures.** In Figure 4, we plot the lateral distributions for the adatom(DA), DA, and its moieties on three differently curved surfaces: the exterior of a (15,15)-CNT nanotube, flat graphene, and the interior of a (15,15)-CNT nanotube. By comparing these distributions to the underlying hexagonal aromatic ring pattern of the carbon surfaces, we can observe how curvature-induced differences in the energy surface roughness influence the placement of these atomic and molecular adsorbates.

First, we consider the lateral distributions of adatom(DA), an atomic adatom with the same mass as DA. Shown in the first row of Figure 4, these distributions clearly display the characteristic hexagonal pattern that corresponds to the centers of the honeycomb structure of the aromatic carbon surface. As the surface curvature changes from convex to concave, the lateral distribution of adatom(DA) gradually becomes more uniform, as expected from the previously noted smoothing of the surface energy as the curvature becomes more negative [23]. Despite the presence of solvating waters in our simulation, the dependence of adatom(DA)’s lateral distribution on the underlying carbon structure and its curvature persists.

In contrast, the lateral distributions of DA’s COM and that of its constituent moieties, as shown in the next four rows of Figure 4, display almost no dependence on the underlying hexagonal carbon structure and we see no clear trend in the distributions with curvature. This lack of structuring and curvature dependence suggests that the underlying surface energy roughness is not a dominant factor in determining the lateral placement of DA, which extends spatially over a region larger than the hexagonal lattice spacing of the underlying carbon surface.

**Figure 5:**
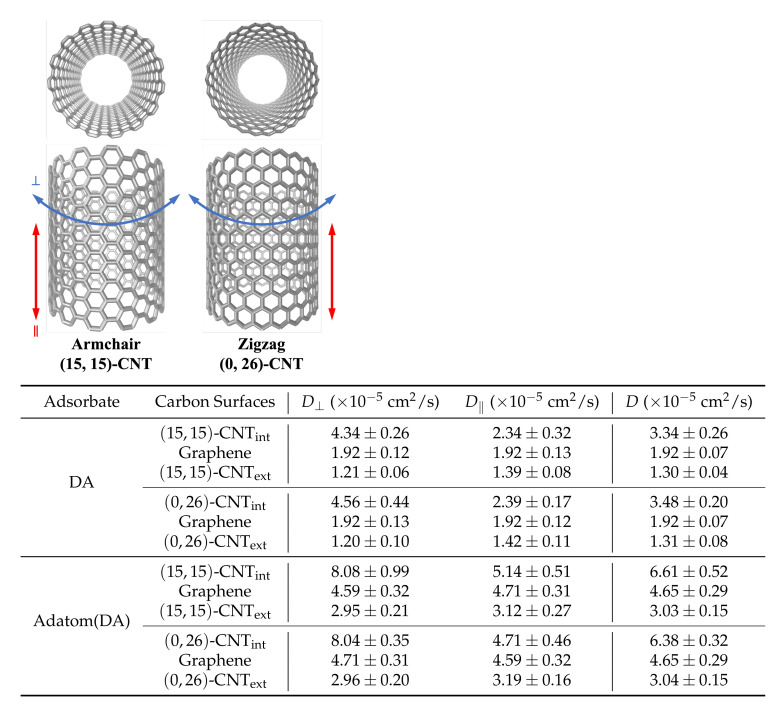
Diffusion coefficients of DA and adatom(DA) on armchair and zigzag CNTs. Armchair and zigzag CNTs are two conformations which describe the carbon atom arrangements along the perpendicular direction. The values of the diffusion constants $D_{⊥}$, $D_{\|}$ and the overall 2D *D* were calculated from the MSDs of the adsorbates on the different surfaces. The 1D diffusion constants on the flat graphene surface along the direction with the same chirality as each CNT direction were chosen for the comparison. Finite system size results are shown here as calculated within the 100 Å long systems.

**Diffusion coefficients for zigzag and armchair CNTs are indistinguishable.** Helicity-dependent diffusion of atomic adsorbates on CNT surfaces has been previously observed in simulations, where different diffusive pathways were observed on armchair and zigzag CNT surfaces due to the surface energy landscapes that emerged upon curving graphene in different directions [23,48]. To probe this effect for our solvated system, we simulated the diffusion of both DA and adatom(DA) on the interior and exterior surfaces of highly curved armchair and zigzag CNTs. Figure 5 shows the two CNT structures with 10 Å radii ((15,15)-CNT and (0,26)-CNT) as well as the $D_{⊥}$, $D_{\|}$, and 2D *D* values obtained from these simulations. The corresponding results on flat graphene are also shown in each case for comparison.

We found no significant difference between the armchair and zigzag diffusion constants in our simulations for either the atomic or molecular DA adsorbates. This result is expected for DA itself, given the insensitivity of its lateral distribution to the underlying hexagonal structure in Figure 4. The shift in the lateral distribution for adatom(DA) with curvature, however, suggests that differences between zigzag and armchair diffusivities are possible within our system. Even so, the results for the two cases are statistically indistinguishable, perhaps due to the dominant influence of surface hydration on adsorbate dynamics in these systems which we found in our prior work on flat graphene [18].

**Curvature dependence of *D* disappears in the absence of solvent.** The negligible influence of the carbon surface’s hexagonal patterning on DA’s lateral distributions in Figure 4 suggests that the differences in *D* between the CNT surfaces of various curvature in Figure 3 and Table 1 do not actually arise from curvature-mediated changes to the energetic interactions between the adsorbate and the surface. The lack of dependence of DA diffusion on CNT helicity in Figure 5 supports this conclusion.

**Figure 6:**
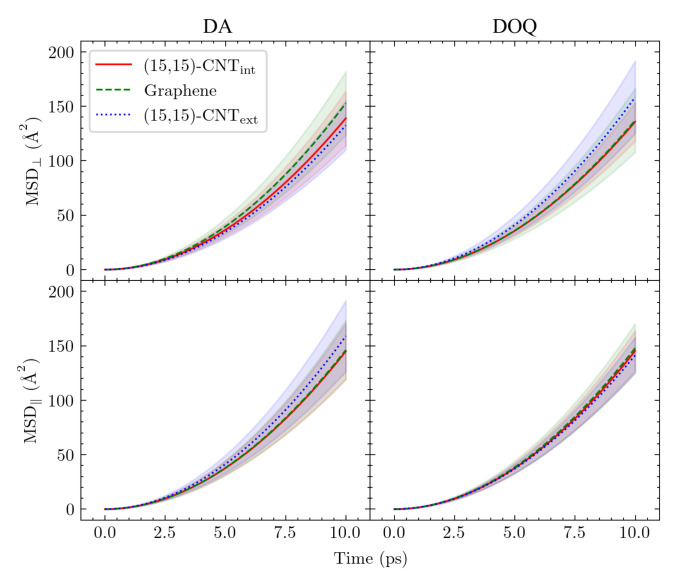
MSDs of DA and DOQ on differently curved carbon:vacuum surfaces. The top and bottom panels show the MSDs as a function of time along two surface directions, ‖ and ⊥, on graphene and on the interior and exterior of the (15,15)-CNT. The left and right panels display the results for adsorbates DA and DOQ, respectively. The lines show the average MSD values, while the shaded regions show the standard deviation in that value across ten trials, with red shading for (15,15)-CNT${}_{\mathrm{int}}$, green shading for flat graphene, and blue shading for (15,15)-CNT${}_{\mathrm{ext}}$.

To isolate the adsorbate–surface interactions and further probe this dependence directly, in Figure 6 we plot MSD${}_{\|}$ and MSD${}_{⊥}$ for DA and DOQ on the carbon surfaces in the absence of solvent. Only the neutral species are simulated due to the lack of charge balance under vacuum conditions. The average MSD results for the adsorbates on flat graphene and on the interior and exterior of the (15,15)-CNT at the vacuum interface are shown plotted with lines, while the noise on each measurement is indicated by the shaded regions.

The resulting curves are not linear over the time regime plotted, indicating that inertial motion lasts for much longer times at the carbon:vacuum interface than at the carbon:water interface. Since the simulations do not allow for carbon surface fluctuations, which would be expected to significantly reduce the timescale of inertial motion decay in the absence of solvent, these MSD curves are only useful as a way to isolate the direct interactions between the adsorbate and the different carbon surface architectures and test their influence on adsorbate diffusion.

The results within each panel show significant overlap of the shaded regions and no observable curvature dependence. In addition, the difference between the diffusivities of DA and DOQ disappears, as expected from our conclusions above regarding the importance of solvent and the effective $R_{H}$ in determining the relative diffusivities of DA, DOQ, and their protonated species [18]. Finally, even the MSD${}_{⊥}$ and MSD${}_{\|}$ curves appear identical, indicating that the differences observed in CNT surface diffusion between the axial and perpendicular directions in Figure 3 and Table 1 are also attributable to solvent effects.

Taken together, these results suggest that the curvature dependence that we observe in the diffusion constants for adsorbed DA and DOQ at the carbon:water surface do not actually arise from curvature-induced changes in the energy surface roughness, as was the case in the prior work on an unsolvated atomic adatom [23]. Instead, we conclude that the curvature-dependence of these molecular adsorbates’ diffusivities arises from a more complex interplay of surface curvature and surface solvation.

### 3.3. Adsorbate Structure Depends on Curvature, Charge, and Solvation

In this section, we investigate in detail the adsorbate’s configuration on the surface and its dependence on curvature, charge, and solvation. First, we examine the vertical placement of DA and DOQ and its constituent moieties above the different carbon surfaces; in particular, we examine the various configurations available to the amine group. Then, we consider the tilt angle of the aromatic ring above the surface and the adsorbate’s orientational alignment with the CNT axis. Finally, we consider how the differently curved surfaces shift the number of water molecules in the first solvation shell around the adsorbate, which will influence the effective hydrodynamic radius, $R_{H}$, and, therefore, the diffusivity.

**Figure 7:**
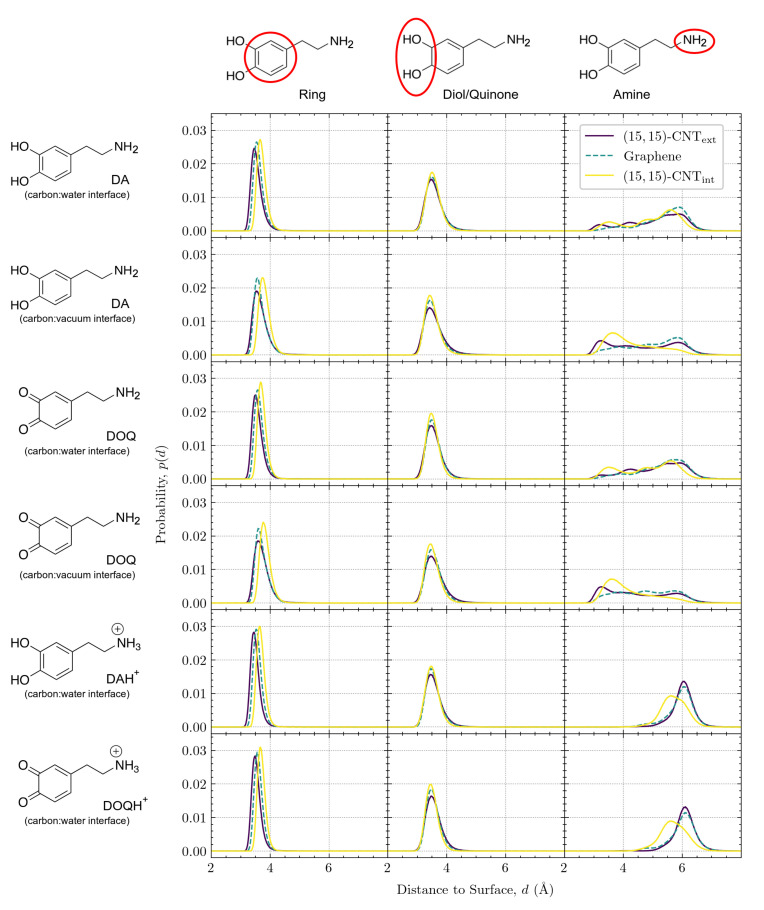
Vertical distributions of DA and DOQ moieties at the carbon:water and carbon:vacuum interfaces. From left to right, three columns show the vertical distributions of the aromatic ring, the diol/quinone moiety, and the amine group, respectively, with *d* representing the distance between that moiety’s COM and the closest point on the surface. From top to bottom, the six rows correspond to DA, DA in vacuum, DOQ, DOQ in vacuum, DAH${}^{+}$, and DOQH${}^{+}$. Colored curves within each subplot indicate the distributions at the flat graphene or the interior and exterior (15,15)-CNT surfaces.

**The distance of the adsorbate above the surface depends on curvature, charge, and solvation.** The vertical distance, *d*, is defined as the distance between the COM of a moiety and its closest point on the carbon surface. Figure 7 displays the vertical distributions for the aromatic ring (left column), the diol/quinone (middle column), and the amine group (right column) on the three surfaces. DA and DOQ distributions are shown at both the carbon:water and carbon:vacuum interfaces, while DAH${}^{+}$ and DOQH${}^{+}$ distributions are only shown at the carbon:water interface.

In the left column of Figure 7, the position of the aromatic ring for all solvated species shifts slightly away from the surface as its curvature changes from convex to flat to concave. Due to the ring’s structural rigidity, its COM can get closer to the surface when adsorbed on the convex exterior of the CNT than when adsorbed to its concave interior, where interactions with the inward-curving walls shift the center of the ring slightly away from its optimal distance on the flat surface. Interestingly, for the two cases of DA and DOQ at the carbon:vacuum surface, the aromatic ring distributions for both the exterior and interior CNT surfaces shift slightly to the right, as compared to the solvated cases. This shift away from the surface indicates the importance of solvation in determining the optimal vertical position for the CNT-adsorbed aromatic rings.

In contrast, the diol/quinone moiety distributions in the middle column of Figure 7 display no shift with curvature, although the peak narrows slightly in all cases as the curvature of the carbon surface changes from convex to flat to concave. The invariance of these peaks, coupled with their location on the edge of the aromatic ring, provide further evidence that the shift in aromatic ring placement with curvature reflects constraints on the optimal surface ring distance due to the curved surface geometry.

Finally, in the right column of Figure 7, we plot the vertical distributions of the amine tail, which is tethered to the aromatic ring through rotatable bonds and can thus adopt a variety of configurations. Our prior work on flat graphene [18] demonstrated that the vertical distribution of the amine group is sensitive to its protonation state, as the positively charged DAH${}^{+}$ and DOQH${}^{+}$ amines can form additional hydrogen bonds with the bulk phase water molecules. These prior observations showed that the neutral amine vertical distributions have three peaks and span a range of about 3–7 Å from the surface, while the positively charged amines have a narrower distribution around a single peak at ≈6 Å from the surface. Similar overall distributions are seen for the CNT exterior and interior surfaces, with a broad, three-peaked distribution for the neutral species and a narrower distribution further from the surface for the charged amines. However, as the curvature changes from the exterior to flat graphene to the interior, the amine distributions are altered, especially for the CNT interior. In addition, we find that the distributions shift closer to the surface for both DA and DOQ at the carbon:vacuum interface as compared to their corresponding distributions at the carbon:water interface.

**Figure 8:**
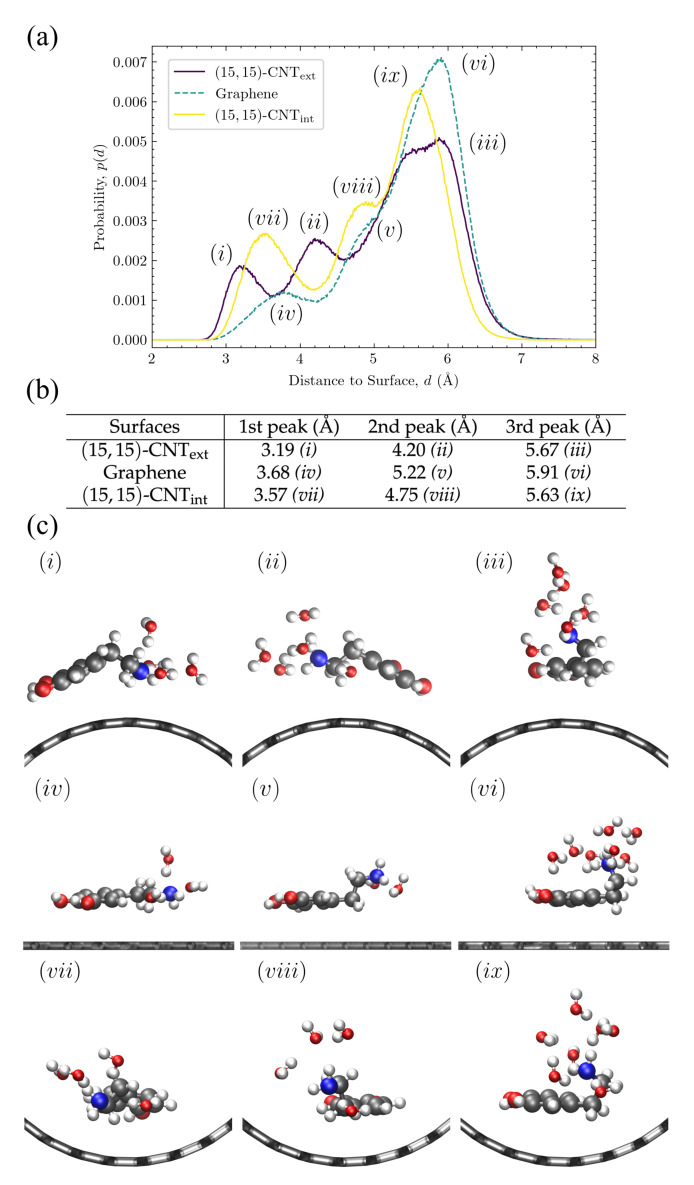
Vertical distributions and configurations of DA at the carbon:water interface. Panel (**a**) shows the vertical distributions of the amine group of DA on flat graphene and on the exterior and interior of a (15,15)-CNT. The three peaks in each distribution are labeled and correspond to the distances shown in panel (**b**) and the sample conformations shown in panel (**c**). Peak positions in (**b**) were obtained from curve-fitting using Gaussian functions. In panel (**c**), only water molecules within 3 Å radius of the nitrogen in the amine group are displayed.

**Amine configurations are highly variable and display significant curvature dependence.** Given the complex variation observed in the amine vertical distributions, in Figure 8, we investigate in more detail these distributions for DA at the carbon:water interface. The three peaks for the CNT${}_{\mathrm{ext}}$, flat graphene, and CNT${}_{\mathrm{int}}$ distributions have been marked with letters in Figure 8a, their positions are listed in the table shown in Figure 8b, and a sample configuration at the characteristic distance within each peak is shown in Figure 8c.

The peaks closest to the surface in Figure 8a*(i, iv, vii)* correspond to configurations in which the amine group is in close contact with the surface. These first amine distribution peaks are observed at 3.1–3.7 Å (see Figure 8b) on all three surfaces, which is close to the sum of the van der Waals (vdW) radii, 3.27 Å, of a carbon with a nitrogen in the OPLS-AA force field [33]. The amine groups in these configurations are closest to the water molecules in the first layer near the surface, as can be seen in the corresponding sample structures in Figure 8c. The first and the second peaks in the density profile of water are observed at ≈3.3 Å and ≈6.2 Å, respectively (see Figure S5). The second set of peaks in the amine distribution *(ii, v, viii)* are observed at 4.2–5.2 Å. The amine groups in these configurations are therefore likely to form hydrogen bonds with both the first and second layers of water molecules, as shown in the sample structures in Figure 8c. Last, the third set of peaks *(iii, vi, ix)* are seen at 5.6–5.9 Å, which is closest to the water molecules in the second layer. The sample configurations for these peaks in Figure 8c show the amine tail stretching up toward the bulk water.

Although the presence of these three peaks persist across the curvatures, their locations shift with curvature, as can be seen in Figure 8a. When the curvature changes from convex (purple) to flat (teal), all three peaks shift rightwards. When the curvature changes from flat to concave (yellow), these three peaks shift back toward the left but to a lesser degree.

To understand the trend in the peak closest to the surface, we consider the three structures shown on the left in Figure 8c. “Tentlike” configurations similar to *(i)* are more likely on the convex surface, where the amine group reaches down toward the carbon surface. Even though the center of the aromatic ring is slightly tilted away from the surface, it remains closer to the surface than it would in a similar configuration on a flat or concave surface. The position of the amine as it points down toward the surface corresponds to the leftmost peak in Figure 8a, at 3.19 Å. The structures *(iv)* and *(vii)* also contain amines quite close to the surface, but given the mismatch between the surface curvature and the tentlike structures of *(i)*, they are not able to get as close, showing a peak distance of 3.68 Å on the flat graphene surface and of 3.57 Å on the convex surface (see Figure 8b).

For the middle peaks, (ii,v,viii), represented by the corresponding structures in the middle column of Figure 8c, the leftward shift is even stronger for DA on the convex surface *(ii)* and represents another version of the “tentlike” structures—one with the same tilted aromatic ring but with the amine group pointing back toward the solvent as in structure (ii) in Figure 8c. In the next section, we discuss the distributions of these aromatic tilt angles and their curvature dependence. On the flat and concave surfaces, the middle peak corresponds to structures where aromatic ring is parallel to the surface and the amine group is rotated away from the surface by one carbon bond in the linker, as in structures *(v)* and *(viii)*.

The third peak from the surface represents the most probably configuration for all curvatures. In these structures, the two linker bonds that connect the plane of the aromatic ring to the amine group are both oriented to extend the amine out away from the surface (see structures (iii,vi,ix) in Figure 8c). The location of this third peak displays the smallest curvature dependency, as can be seen in the relatively small range in the most probable distances listed in Figure 8b, third column. Although the structures shown in Figure 8c only include the neutral DA species, this third peak is the only one observed for the positively charged species, DAH${}^{+}$ and DOQH${}^{+}$ (see the last two rows of Figure 7, right-most column). This result indicates that, when protonated, the amine group remains fully extended into the solvent for all curvatures, similar to structures (iii,vi,ix). These structures also aid in the interpretation of the amine distributions for DA and DOQ at the carbon:vacuum interface in Figure 7 as well. As compared to the same amine distance distributions at the carbon:water interface, the peak locations remain unchanged, but the relative peak heights shift, indicating that configurations with the amine extending away from the surface are significantly less probable in the absence of solvent.

**Figure 9:**
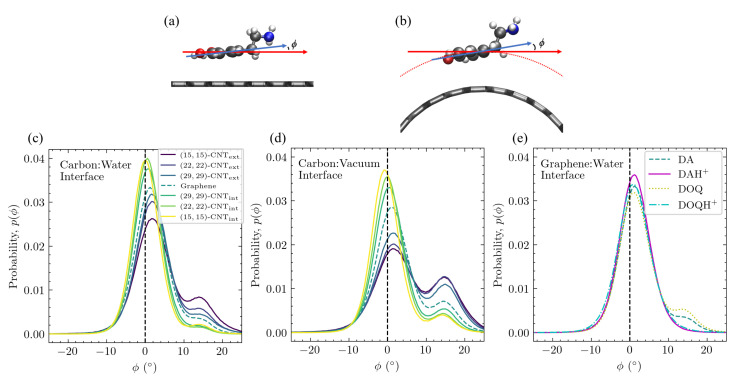
Tilt angle distributions of DA and other adsorbates on differently curved and solvated CNT and graphene surfaces. The tilt angle ϕ is defined as shown in (**a**,**b**) between the C2–C7 vector (blue arrows) and a vector tangent to the surface at the midpoint of the C2–C7 vector (red arrows). ϕ distributions for DA on differently curved surfaces, plotted as histograms with a binwidth of 0.36${}^{\circ }$, are shown in (**c**) at the carbon:water interface and in (**d**) at the carbon:vacuum interface. The results for DA, DOQ, and their protonated counterparts are shown in (**e**) on solvated flat graphene.

**The aromatic ring’s tilt angle above the surface depends on curvature, charge, and solvation.** The distributions of the tilt angle between the aromatic ring and the surface are shown in Figure 9 for DA on differently curved carbon surfaces at both the carbon:water (Figure 9c) and carbon:vacuum (Figure 9d) interfaces. In addition, the tilt distributions of DA, DOQ, and their protonated species are shown for flat graphene at the aqueous interface in Figure 9e.

When adsorbed on all carbon surfaces, DA primarily adopts configurations in which its aromatic ring is parallel to the surface, as seen from the dominant peak, which is close to $\phi =0^{\circ }$ in all cases. This configuration maximizes the π–π interactions and is seen in most of the structures shown in Figure 8c. However, a second, asymmetric peak is observed in a subset of the cases at $\phi \approx 15^{\circ }$ and corresponds to the tentlike configurations seen in structures *(i)* and *(ii)* in Figure 8c. The relative probability of these two tilt angles clearly depends on the surface curvature—the peak at $\phi \approx 0^{\circ }$ is strongest on the most concave surface, whereas the peak at $\phi \approx 15^{\circ }$ is strongest on the most convex surface.

The tilt angle distributions also display a clear dependence on charge, as can be seen on solvated flat graphene in Figure 9e, where there is substantial probability around $\phi \approx 15^{\circ }$ for DA and DOQ but no such density for DAH${}^{+}$ and DOQH${}^{+}$. From the amine group distributions for these positively charged species in Figure 7, we know that they adopt configurations in which the amine group stretches out into the bulk water, which precludes the more tilted tentlike structures like *(i)* and *(ii)* in Figure 8c.

The tilt angle distribution also depends on solvation. As can be seen in Figure 9d, the curvature-dependence observed at the carbon:water surface is also present at the carbon:vacuum surface. However, the population of the tilted configuration increases in all cases, which corresponds well to the shift in the amine distance distribution to values that are closer to the surface for the carbon:vacuum surfaces in Figure 7. Similar results were obtained for DOQ at the carbon:vacuum interface, see Figure S6a.

**Figure 10:**
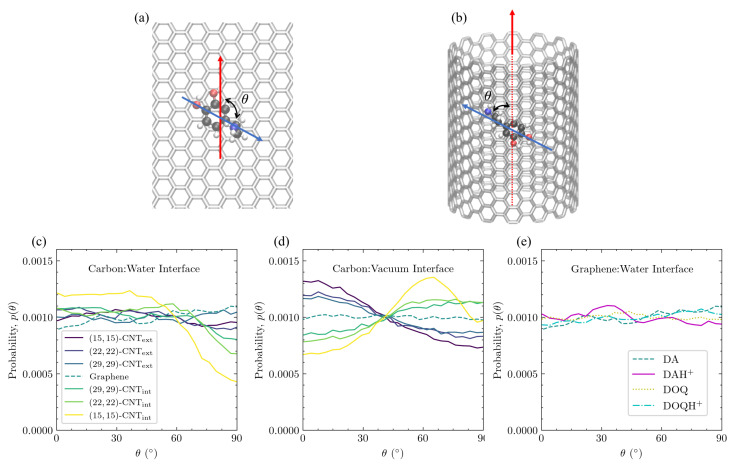
Orientational alignment of DA and other adsorbates with the CNT axis direction on differently curved and solvated CNT and graphene surfaces. The orientational angle, θ, is defined as the angle between the C2–C7 vector (blue arrows) and the CNT axis direction (red arrows), as shown in (**a**,**b**). θ distributions of DA on differently curved surfaces, plotted as histograms with a binwidth of 3.6${}^{\circ }$, are shown in (**c**) for the carbon:water interface and in (**d**) for the carbon:vacuum interface. The same results for DA, DOQ, and their protonated counterparts are shown in (**e**) on solvated flat graphene. θ has a range of $\left[0,180^{\circ }\right]$; however, the results are wrapped so that $p\left(\theta \right)=p\left(180^{\circ }-\theta \right)$, for all $\theta >90^{\circ }$, due to the symmetry of the system.

**Adsorbate alignment with CNT axis also depends on curvature and solvation.** In Figure 10, the orientational alignment of DA with the axis of the CNT, as defined by θ in Figure 10a,b, is shown on differently curved carbon surfaces at the carbon:water interface (Figure 10c) and at the carbon:vacuum interface (Figure 10d). The θ distributions of DA, DOQ, and their protonated species are also shown for flat graphene at the aqueous interface in Figure 10e.

At the carbon:water interface, the θ distribution of DA is uniform on flat graphene and on the exterior of the CNTs, as can be seen in Figure 10c. In addition, charge does not seem to influence this orientation for the solvated flat graphene case shown in Figure 10e. Even the flat graphene case at the carbon:vacuum interface in Figure 10d shows no change in the probability with θ. This invariance of the probability of a given θ orientation on flat graphene is to be expected, given the lack of curvature to break the symmetries present in the flat graphene case as well as the lack of significant lateral distribution patterning in Figure 4.

In contrast, on the solvated CNT interior in Figure 10c, there is a marked decrease in the orientational probability as θ approaches $90^{\circ }$, and the effect becomes more dramatic as the concavity increases. These highly curved interior surfaces favor orientations where the longest axis of DA is oriented along the CNT axis ($\theta =0\pm 40^{\circ }$). This orientational preference is linked to the rightward shift in the aromatic ring’s vertical distribution as the surface changes from flat to concave, as shown in Figure 7a. In configurations where DA is not aligned with the CNT axis, its interactions with the inward-curving walls will force the center of the ring slightly away from its optimal distance above the surface. The data obtained from ten trajectories show that, on the solvated interior of the (15,15)-CNT nanotube, where this orientational preference is strongest, the average vertical distance of the ring’s COM for all configurations in which DA is closely aligned with the CNT axis ($|\theta |<5^{\circ }$) is 3.66±0.15 Å, whereas the average vertical distance for the configurations where DA is perpendicularly aligned to the CNT axis ($85^{\circ }<\left|\theta \right|<95^{\circ }$) is 3.90±0.17 Å.

The θ distribution is entirely different at the carbon:vacuum interface, however. Orientations aligned with the CNT axis are disfavored on the CNT interior, and the most favorable orientation on the CNT interior shifts to ≈65${}^{\circ }$. At the same time, orientations aligned with the CNT axis are favored on the CNT exterior. Both trends grow stronger with increased curvature, and the same trends were observed for DOQ at the carbon:vacuum interface (Figure S6b).

**Figure 11:**
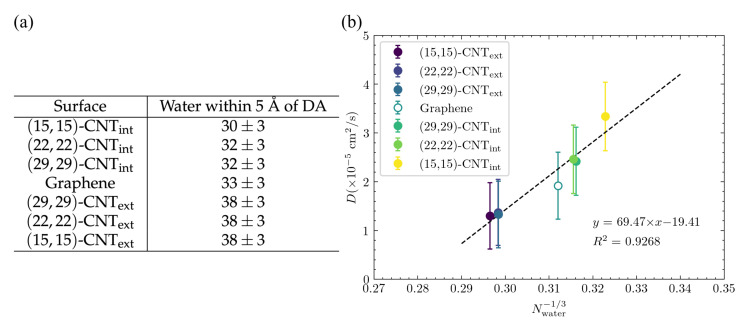
Correspondence between CNT surface, solvating waters, and *D*. (**a**) The number of waters in the first solvation shell around DA are calculated across the different surfaces. For a given water molecule, its distance to DA is the shortest distance between its oxygen atom and any the atoms of DA. Only the water molecules that are on the same side of the surface as DA are counted. $N_{\mathrm{water}}$ and its statistical errors were computed from 10 trajectories. (**b**) The diffusion constants, *D*, from Figure 3c are plotted vs. $N_{\mathrm{water}}^{-1/3}$ for DA on a range of differently curved surfaces. A linear fit line is shown here along with the coefficient of determination, $R^{2}$.

**Adsorbate solvation shell depends on curvature and influences $R_{H}$.** According to the Stokes–Einstein equation, the diffusion constant, $D\propto (1/R_{H})$, where $R_{H}$ is the effective hydrodynamic radius, which depends on the magnitude of attractions between the diffusing particle and the nearby solvent molecules. In the case of a particle adsorbed to a surface, solvation is necessarily limited by the presence and geometry of that surface. Although the Stokes–Einstein relation cannot be directly applied in that situation, it does provide a way to think about the influence of the degree of solvation on diffusion, as the magnitude of any favorable interactions between the particle and nearby solvent will influence the particle’s effective hydrodynamic radius, $R_{H}$. To investigate this effect, we calculated the number of solvating water molecules within the first water shell around the DA or DOQ atoms for each surface architecture. A distance of 5 Å was chosen as the cutoff of that first water shell based on the distribution shown in Figure S5. The results are shown in Figure 11a and display a clear trend from fewest solvating waters on the smallest CNT’s interior to the most solvating waters on the smallest CNT’s exterior—as expected given the geometric constraints of the surface. This trend matches that seen in the diffusion constants on different surface curvatures, as seen in Figure 3c. To determine how well this solvation effect can explain the trend in diffusivities, we plotted in Figure 11b the diffusivities from Figure 3c vs. ${\left(N_{\mathrm{water}}\right)}^{-1/3}$, where $N_{\mathrm{water}}$ is the number of waters within 5 Å of a DA atom, since $D\propto (1/R_{H})$, and $R_{H}$ is roughly $\propto {\left(N_{\mathrm{water}}\right)}^{1/3}$. The correspondence is quite strong, and this effect is even able to explain the overlapping values seen in the diffusivities even as curvature steadily changes for the CNT exteriors and for the (22,22)-CNT${}_{\mathrm{int}}$ and (29,29)-CNT${}_{\mathrm{int}}$ cases. Since there is a clear geometric trend across these different diameter CNTs, the fact that the number of solvating waters is the same implies that a change in the adsorbate structures, as documented above, must compensate for that change in a way that maintains a similar degree of solvation.

Overall, we find here that the vertical placement of the adsorbate and its moieties above the carbon surface, as well as its tilt angle and alignment with the CNT axis depend in a complex manner on curvature, solvation, and charge. In addition, the degree of DA solvation varies with curvature and can explain much of the trend observed as *D* varies across curvatures.

### 3.4. DA Localizes and Diffuses within a CNT Groove

Since a pair of aligned CNTs is the simplest multi-CNT structure expected within CNT-based material, we also investigated how DA behaves on a groove surface, both for the solvated and vacuum cases.

In all the simulations, DA localized to the groove between the two CNTs within 2–8 ns and remained there. Given this strong structural preference, we ran each simulations for at least 5 ns after it found its way to the groove. All results presented in this section were obtained from the portions of the trajectories where DA is within the CNT groove.

**Figure 12:**
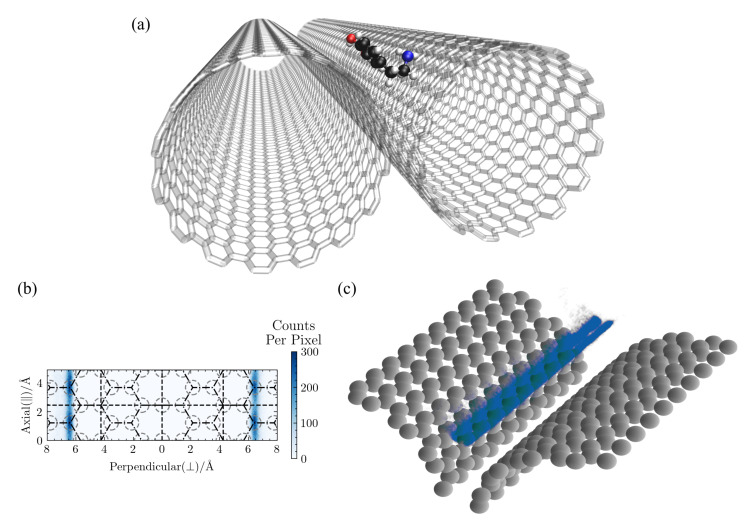
Spatial distributions of DA in a solvated CNT groove. The COM coordinates of DA within a solvated CNT groove formed by two parallel CNT, as seen in a typical configuration shown in (**a**), are plotted here in both (**b**) 2D and (**c**) 3D. The CNT groove is constructed of two parallel, 100 Å (15,15)-CNTs. In the 2D distribution plot in (**b**), locations along the axial direction were wrapped into two unit cells. The gray dashed circles represent the location of the surface carbon atoms, and the black dashed line in the middle at ⊥=0 Å represents the location on the CNT circumference where the distance between the two CNTs is smallest. The distribution density in region to the left of that dashed line results from configurations where the adsorbate is closest to the CNT on the left, while the density to the right results from configurations where the adsorbate is closest to the CNT on the right. In the 3D distribution in (**c**), the COM coordinates along the axial direction were wrapped into ten unit cells for plotting.

A typical configuration from a groove simulation is shown in Figure 12a, the lateral distribution of DA is shown in Figure 12b, and the 3D distribution is shown in Figure 12c. There is a slight dependence on the underlying hexagonal structure in the lateral density distribution in Figure 12b, but only the axial direction, as the adsorbate’s location around the circumference is determined by the optimal distance from the other CNT surface, as can be seen in Figure 12c. Note that any lateral patterning will depend on the degree to which the neighboring CNTs are in register. There is a clear separation in the 3D density plot between configurations with DA adsorbed to one CNT surface vs. the other. Jumps between the two CNT surfaces are rare in the solvated case (1.9±0.4 ns${}^{-1}$), but were more frequently for DA adsorbed at the carbon:vacuum interface (49.2±7.4 ns${}^{-1}$). Jump trajectories across both the carbon:water and carbon:vacuum CNT grooves can be seen in Figure S7.

**Figure 13:**
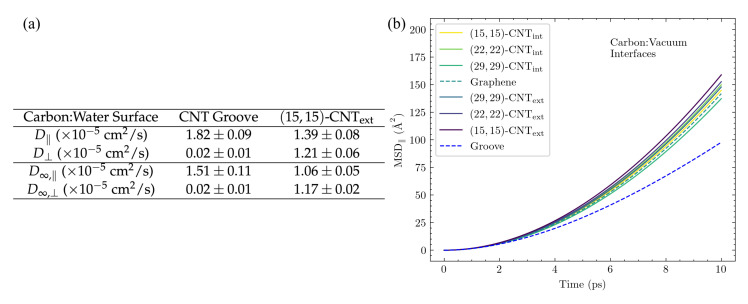
Diffusion coefficients of DA within a CNT groove. The CNT groove is constructed of two 100 Å long, aligned, (15,15)-CNTs. (**a**) Diffusion constants for the solvated CNT groove and a solvated (15,15)-CNT${}_{\mathrm{ext}}$ surface are shown here, both from the 100 Å simulation directly (top rows) and from the infinite-system size extrapolation (bottom rows). $D_{\|}$ is the 1D diffusion coefficient for motion along the CNT groove and axis, and $D_{⊥}$ is the 1D diffusion coefficient around the CNT circumference. (**b**) The axial mean squared displacement of DA is plotted for various surfaces at the carbon:vacuum interface. These MSD results are taken directly from simulations done in the vacuum on 100 Å CNTs and 100×100 Å${}^{2}$ graphene.

Figure 13a compares $D_{\|}$ and $D_{⊥}$ for DA in the solvated groove to the same values for DA on the exterior of a single (15,15)-CNT, since the groove is constructed of two aligned (15,15)-CNTs. Results directly obtained from the 100 Å-length CNT groove system are shown in the top section of the table, while the extrapolation to the infinite CNT groove is shown at the bottom. Importantly, the observed trends hold for both the finite size results and the infinite size extrapolations. As expected, $D_{⊥}$ drops to almost zero when DA remains in the groove. In contrast, DA’s diffusivity along the groove, $D_{\|}$, is significantly faster than the corresponding axial diffusivity on the exterior surface of a single CNT. We then calculated $N_{\mathrm{water}}$ for DA in the groove and found 26±3 waters within 5 Å. This value is lower than those reported in Figure 11a for the other surface structures and explains the faster diffusion within the solvated groove.

Interestingly, this trend is reversed for DA’s diffusivity in the groove at the carbon:vacuum interface. Figure 13b shows the comparison of the axial MSD of DA in the groove to that of DA on other CNT and graphene surfaces, all at the carbon:vacuum interface. Without solvent, displacement along the groove is reduced as compared to that on any other surface, which can be readily explained by the presence of two variegated surfaces that can impact DA’s inertial motion rather than just one.

The results for the diffusion of all four adsorbate species within the 100 Å CNT groove are shown in Table 3. The previously observed trends between oxidized and reduced species (oxidized diffuses more rapidly) and between protonated and neutral species (neutral diffuses more rapidly) both hold within the groove architecture.

## 4. Conclusions

Overall, we find that DA and DOQ rapidly diffuse on the surface of pristine CNTs, just as they do on the flat graphene surface [18]. Diffusion on a single CNT is rapid both along the CNT axis and around its circumference. This observation corresponds to results from Kim et al., who developed a continuum model on the µm scale to demonstrate the catalytic activity of the exterior sidewall of individual CNTs. The model shows evidence that the entire length of the CNT is uniformly accessible to the electrochemically active analytes, which matched their spatially resolved scanning electrochemical microscopy results [12].

At the same time, we find that the adsorbate diffusivity also depends on the CNT curvature. We observed enhanced adsorbate diffusion as the surface changes from convex to flat to concave. Although this trend is similar to that observed previously for atomic adsorbates on CNT surfaces [23,24], its origin differs. In our study, where molecular adsorbates are diffusing on a solvated surface, the curvature-dependent diffusion cannot be attributed to changes in the underlying surface energy roughness with curvature, as the lateral distributions of the molecular adsorbates do not depend on curvature. In addition, we find that the diffusion constants on the zigzag and armchair CNTs are indistinguishable. Finally, in the absence of solvent, the curvature dependence disappears.

Why, then, does adsorbate diffusivity change with curvature? First and foremost, the degree of solvation depends upon the surface geometry and will influence the adsorbate’s effective hydrodynamic radius, $R_{H}$, and the Stokes–Einstein equation, although not quantitatively applicable here, tells us that the diffusion constant goes as 1/$R_{H}$. Second, we also observe systematic shifts in the adsorbate tilt angle and axial orientation with surface curvature, which are also influenced by solvation. Last, multiple studies have shown changes in solvent dynamics within a CNT [25,27,28,30,31], which could influence adsorbate dynamics in our simulation. While most of these effects are for CNTs with diameters significantly smaller than ours, enhancements in solvent diffusion have been seen at the interior of narrow CNTs and are more dramatic close to the CNT surface [25,27]. We also note that we observe more significant finite size effects in the CNT interior systems (see Figure S4); however, the observed trend with curvature holds even when the correction is made to an infinitely long CNT system (see Table 1).

Directional diffusion of adsorbates on the surface of CNTs is of general interest for several applications [23,41,49,50,51]. Prior work studying adatom diffusion on the CNT surface noted substantial differences between diffusion pathways on armchair and zigzag CNTs, with the adatom diffusing exclusively around the zigzag CNT’s circumference, while on the armchair CNT, the adatom moved axially as well [23]. It may be concluded that CNT helicity could play an important role in determining mass transport on these nanoscale surfaces. However, we found no such effect on the surface of a single CNT in our system; the larger molecular structure of DA and DOQ decreased the importance of the direction of strain in the underlying CNT hexagonal surface. More importantly, solvation at room temperature removes the helicity dependence for even the adatom(DA) in our simulations, despite the fact that its lateral placement depends on the direction of curvature (see Figure 4, top row). As a result, we do not expect CNT helicity to play an important role in determining the directionality of diffusive transport for adsorbates on solvated surfaces at room temperature. At the same time, we did observe significant changes in the direction of diffusion for DA at the groove junction between two aligned CNTs. Once the adsorbate encountered the groove, it stayed there and subsequent diffusion was restricted to the axial direction. This directional effect could therefore have a significant impact on mass transport within CNT-based nanomaterials.

Although the work in this paper focuses on single-walled CNTs and graphene, we expect that our key findings can be extended to other carbon nanostructures. On multiwalled CNTs, we anticipate dopamine structures, diffusion timescales, and curvature trends that are similar to single-walled CNTs with the same interface curvature, since we found previously that dopamine diffusion on one layer of pristine graphene was indistinguishable from that on a triple layer of graphene [18]. Dopamine diffusion on the exterior of fullerenes will also be similar to that on the exterior of the highly curved CNTs. However, the slow “hopping” rate we observed from one CNT surface to another—even when both surfaces share an extended edge where they are in close proximity—makes it clear that adsorption of DA to fullerene, or other 0D nanostructures, would localize DA for long timescales. Thus, we expect that some fraction of the mass transport of adsorbed DA would be arrested on a composite surface that incorporates fullerenes. Diffusion of molecular adsorbates on extended and 3D carbon surfaces are likely to differ significantly from our observations on CNTs, as these structures may have highly confined waters, where dynamics are known to differ significantly from that of bulk water [27,28,29]. In addition, we have found that the degree of adsorbate solvation at the carbon:aqueous interface, coupled with the local water dynamics, is essential for determining adsorbate diffusivities across a range of molecular carbon surfaces.

CNTs have become common materials for electrochemical sensors but the diffusion of common analytes, such as dopamine, has not been understood on their surface. Here, we find that diffusion is fast on CNTs, about as fast as on flat graphene. However, CNT-based electrodes are not made of single CNTs, and many electrodes consist of aligned CNT materials, such as CNT forests or CNT yarns [3,4,5]. Thus, modeling a CNT groove shows that interactions between CNTs also affect dopamine dynamics. The CNT groove provides directionality for movement and localizes the dopamine on one part of the CNT. In future studies, we could introduce a voltage and examine electrochemistry. We expect that the observed shifts in tilt angle, axial orientation, and analyte–surface distance that occur upon changes to CNT curvature will be important for electron transfer. These are the first studies of dopamine diffusion on CNT electrodes and provide foundational information about the surface structure and dynamics of dopamine adsorbates on electrode surfaces.

## Figures and Tables

**Table 1:** Diffusion coefficients extrapolated to the infinite system sizes. For a subset of the carbon surfaces, diffusion constants for the infinite system sizes, $D_{\infty }$, were extrapolated from a series of differently sized finite simulations. The extrapolation was done to correct for unphysical effects that arise from the necessarily finite simulation sizes, and the $D_{\infty }$ values thus represent the actual diffusivities expected within the larger physical systems. See SI and Figure S4 for details.

Adsorbate	Carbon Surfaces	$D_{\infty ,⊥}$ ($\times 10^{-5}$ cm${}^{2}$/s)	$D_{\infty ,\|}$ ($\times 10^{-5}$ cm${}^{2}$/s)	$D_{\infty }$ ($\times 10^{-5}$ cm${}^{2}$/s)
DA	(15,15)-CNT${}_{\mathrm{int}}$	3.50±0.15	1.41±0.19	2.45±0.13
	Flat Graphene	1.27±0.07	1.22±0.10	1.24±0.06
	(15,15)-CNT${}_{\mathrm{ext}}$	1.17±0.02	1.06±0.05	1.12±0.03

**Table 2:** 2D diffusion coefficients of DA, DOQ, and their protonated counterparts. The values of the overall 2D diffusion constant, *D*, were calculated from the MSDs of the adsorbates on the interior of the armchair (15,15)-CNT, flat graphene, and the exterior of the armchair (15,15)-CNT. Finite system size results are shown here as calculated within the ≈100 Å long systems.

*D* ($\times 10^{-5}$ cm${}^{2}$/s)	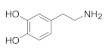	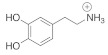	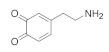	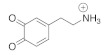
	DA	DAH${}^{+}$	DOQ	DOQH${}^{+}$
(15,15)-CNT_int_	3.34±0.26	3.24±0.21	3.72±0.29	3.65±0.35
Graphene	1.92±0.07	1.74±0.07	2.29±0.16	1.95±0.14
(15,15)-CNT_ext_	1.30±0.04	1.20±0.06	1.53±0.10	1.37±0.05

**Table 3:** Diffusion coefficients of DA, DOQ, DAH${}^{+}$, and DOQH${}^{+}$ in a solvated CNT groove. The CNT groove results shown here are reported directly from the finite 100 Å-long CNT simulations.

Adsorbate	$D_{\|}$ ($\times 10^{-5}$ cm${}^{2}$/s)	$D_{⊥}$ ($\times 10^{-5}$ cm${}^{2}$/s)
DA	1.82±0.09	0.02±0.01
DOQ	1.98±0.17	0.04±0.03
DAH${}^{+}$	1.60±0.10	0.02±0.01
DOQH${}^{+}$	1.63±0.05	0.02±0.00

## Data Availability

The data presented in this study, along with submission and analysis scripts, are openly available on GitHub at https://github.com/dubayresearchgroup/DopaDiff.

## Supplementary Materials

The following supporting information can be downloaded at: https://www.mdpi.com/article/10.3390/molecules27123768/s1. References [52,53,54] are cited in the supplementary materials.
