# Supplementary material for: Structure and Dynamics of Adsorbed Dopamine on Solvated Carbon Nanotubes and in a CNT Groove
Source: Molecules. 2022 Jun 11;27(12):3768. doi: 10.3390/molecules27123768 (PMC9228466; doi:10.3390/molecules27123768)
Supplement: Supplementary file 1 [file molecules-27-03768-s001.zip › molecules-1734869-supplementary.pdf]

# Supplemental Information for "Structure and Dynamics of Adsorbed Dopamine on Solvated Carbon Nanotubes and in a CNT Groove"

Qizhang Jia, B. Jill Venton, Kateri H. DuBay*

*Department of Chemistry, University of Virginia, Charlottesville, Virginia 22904*

E-mail: dubay@virginia.edu

## 1 Simulation Details

In this study, the single-walled CNTs have two types of helicities and three different diameters, as listed in Table S1. The chiral index $(n, m)$ defines the wrapping vector or chiral vector of a CNT, $\vec{C_h} = n\vec{a_1} + m\vec{a_2}$, where $\vec{a_1}$ and $\vec{a_2}$ vectors are the basis of graphene Bravais lattice [45]. In the $(n, m)$ notion, $n = m$ for the armchair CNTs, while $n = 0$ for the zigzag CNTs [45,53]. Both CNTs helicities were constructed for three CNT sizes, with diameters of approximately 20, 30 and 40 Å, see Table S1.

The modeled systems first underwent an equilibration run in the $NPT$ ensemble at 300K and 1 atm using a Nosé-Hoover thermostat and barostat. In order to maintain the structural integrity of the infinite carbon surfaces in equilibration runs containing a CNT, the simulation box was dilated and contracted in the $x$ and $y$ directions in a coupled manner, so as not to stretch or shrink the CNTs along its axial direction. Similarly, in equilibrium runs with a graphene surface, the simulation box was dilated and contracted only along the $z$ direction.

The solvated production runs were performed in the $NVT$ ensemble using a Nosé-Hoover thermostat at 300K for 7 ns from ten randomly generated initial configurations. For the solvated simulations with a groove surface, the $NVT$ production runs were performed for an additional 7 ns to ensure sufficient data once the adsorbate localized to the groove. For the vacuum simulations, the $NVE$ ensemble was used and simulations began from ten different equilibrated configurations gathered from the set-up runs. Equations of motion were integrated using a standard velocity-Verlet algorithm with a time step of 1 fs. Data from the last 5 ns of all simulated trajectories were recorded in intervals of 100 ps and used in the calculation of the diffusion coefficients, i.e., nanoseconds 2-7 for graphene and single CNT simulations and nanoseconds 9-14 for CNT groove simulations.

Details regarding the equilibration process and the partial charge calculations can be found in Ref. [18], as can discussions of the effects of holding the carbon surface fixed and using only

Table S1: **CNT structural details.** The exact types, helicities, diameters, and lengths of the modeled CNTs are shown here. CNT diameters were calculated as described in Ref. [52].

| Type $(n, m)$-CNT | Helicity | Diameter (Å) | Length (Å) |
|---|---|---|---|
| $(15, 15)$-CNT | | 20.311 | |
| $(22, 22)$-CNT | Armchair | 29.790 | 100.698 |
| $(29, 29)$-CNT | | 39.269 | |
| $(0, 26)$-CNT | | 20.326 | |
| $(0, 38)$-CNT | Zigzag | 29.708 | 102.096 |
| $(0, 51)$-CNT | | 39.871 | |

a single layer of graphene for the graphene surface.

## 2 Data Analysis

**Projecting adsorbate trajectories onto the surface.** Since all adsorbates in our simulations quickly adsorbed onto the surfaces and never desorbed, we projected their center-of-masses (COMs) onto the carbon surface in order to calculate their on-surface, 2D trajectories.

In order to quantify and compare the surface motion of diffusing species on the curved CNTs with their diffusion on the flat graphene surface, the Cartesian coordinates, $\{x, y, z\}$, were transformed to cylindrical coordinates, $\{r, \theta, z\}$, according to

$$\begin{aligned} r &= \sqrt{(x - x_{\text{CNT}})^2 + (y - y_{\text{CNT}})^2}, \\ \theta &= \text{atan2}(\frac{y - y_{\text{CNT}}}{x - x_{\text{CNT}}}), \\ z &= z, \end{aligned} \tag{S1}$$

where $(x_{\text{CNT}}, y_{\text{CNT}})$ denotes the location of the CNT axis, and `atan2` is the two-argument arctangent function, which returns a corrected and unambiguous arctangent angle within the range $[-\pi, \pi]$. This transformation is illustrated in Fig. S1. In essence, the cylindrical coordinate transformation cuts the nanotube surface along its axis and unrolls it to form a plane consisting of two dimensions, $r$ and $\theta$.

However, when performing the transformation shown in Eqn. S1, the unambiguous two-argument arctangent function, `atan2`, results in discontinuities of the azimuthal angle $\theta$ near $-\pi$ and $\pi$. To reflect the continuous motion of the diffusing adsorbate on the cylindrical nanotube surface, we used a threshold angular displacement of $\theta_{\text{threshold}} = 0.1\pi = 18°$ to indicate a break in the continuous trajectories of $\theta$ where the adsorbate moved across the discontinuous $-\pi$ and $\pi$ positions. Specifically, if the angular displacement between any two consecutive frames in time (separated by 0.01 ps) exceeded $\theta_{\text{threshold}}$, the rest of the $\theta$ trajectory is shifted by $\pm 360°$ to restore the continuity in the azimuthal coordinate. The raw and corrected, continuous trajectories in the $\theta$ variable are shown in Fig. S2a. Here, the raw blue trajectory computed directly from the two-argument arctangent function (`atan2`) has several discontinuous points, while the corrected green trajectory is continuous, reflecting the continuous motion of the diffusing adsorbate around the cylindrical nanotube's circumference. The choice of $\theta_{\text{threshold}} = 18°$ is justified by the distributions of $\theta$ displacements observed between two consecutive structural frames (separated by 0.01 ps), as shown in Fig. S2b.

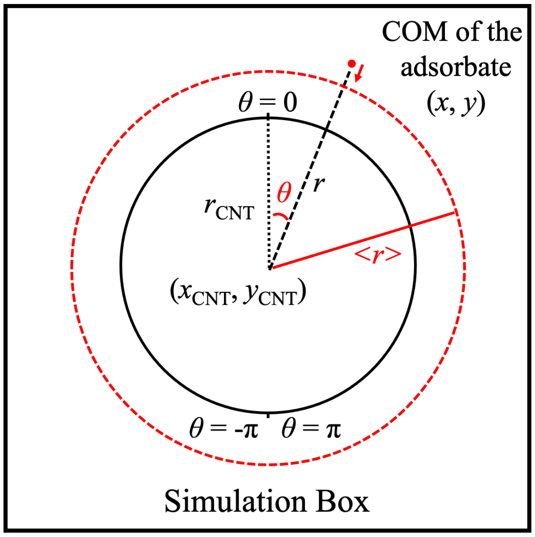


Figure S1: **Schematic of the cylindrical coordinate transformation.** This figure illustrates the cross-section of a CNT and the variables used in the cylindrical coordinate transformation. $(x, y, z)$ are the diffusing adsorbate's COM Cartesian coordinates, and $(r, \theta, z)$ are the transformed cylindrical coordinates using the Eqn. S1. $(x_{\text{CNT}}, y_{\text{CNT}})$ denotes the location of the CNT axis, $r_{\text{CNT}}$ is the CNT radius, and $\langle r \rangle$ is the ensemble average radial coordinate of the adsorbate during the simulation. The red dashed circle is an imaginary cylindrical surface onto which the COM of the adsorbate is projected during the MSD calculation. Note: the simulation box and CNT are not drawn to scale.

**Calculating Diffusion Coefficients.** The Einstein relation associates the diffusion coefficient, $D$, with the slope of the linear regime of a particle's MSD by

$$\langle \Delta R^2(t) \rangle = 2dDt, \tag{S2}$$

where $\langle \Delta R^2(t) \rangle$ denotes the ensemble average of the MSD over time, $t$, and $d$ is the diffu-

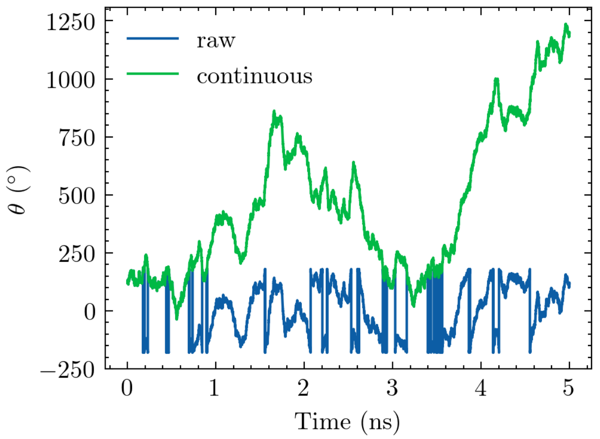


]
(a)

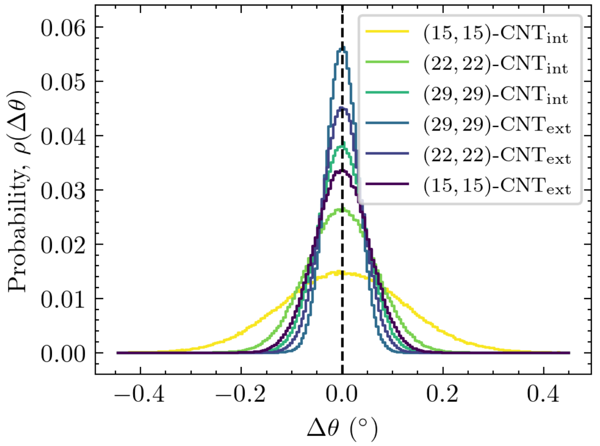


]
(b)

Figure S2: **Azimuthal continuity within CNT trajectories.** When performing the cylindrical coordinate transformation (Eqn. S1), the two-argument arctangent function, `atan2`, results in a discontinuity of the azimuthal angle, $\theta$, between $-\pi$ and $\pi$. We used a threshold angular displacement of $\theta_{\text{threshold}} = 0.1\pi = 18°$ to shift $\theta$ at this crossover point to recover the continuous trajectories. (a) A representative 5.0 ns raw (blue) and continuous (green) azimuthal angle, $\theta$, trajectory is shown here from a 300K simulation of DA on the interior of a (15, 15)-CNT nanotube. (b) The distribution of DA's angular displacement, $\Delta\theta$, between two consecutive frames of recording is plotted here for various CNT surfaces. Each recording frame is separated by 0.01 ps. The maximum displacement of the diffusing adsorbate in between two consecutive frames remains well below the displacement threshold, $\theta_{\text{threshold}} = 18°$, validating the approach.

sional dimensionality. From this equation, $D$ can be calculated in the diffusive regime by linearly fitting the MSD after the inertial motion dissipates.

Eqn. S2 describes the Einstein relation in the Cartesian coordinates, and Castro-Villarreal *et al.* expanded this relation to the Brownian motion of a free particle on a $d$-dimensional Riemannian geometry [54]. The mean-squared geodesic distance (MSGD, $s$) of the diffusing particle as a function of time is related to its diffusion coefficient, $D$, by

$$\langle s^2(t)\rangle = 2dDt - \frac{2}{3}R_g(Dt)^2 + \frac{4}{45}\frac{d-3}{d(d-1)}R_g^2(Dt)^3 + \mathcal{O}(t^4), \tag{S3}$$

where $\mathcal{O}(t^4)$ includes $t^4$ and higher order terms. In this equation, the Riemann curvature tensor, $R_g$, reflects the surface geometry. $R_g > 0$ for spherical curvature, $R_g < 0$ for hyperboloidal curvature, and in the special Euclidian geometry case, when $R_g = 0$, MSGD becomes the conventional MSD, and Eqn. S3 reduces to the Einstein relation shown in Eqn. S2. Since the curved CNT cylindrical surface has an Euclidian geometry where $R_g = 0$, the diffusion coefficients are computed from our simulations using

$$D = \frac{\langle \Delta R^2(t)\rangle}{2dt}, \tag{S4}$$

where $\langle \Delta R^2(t)\rangle$ denotes the ensemble average of the MSD as a function of time, $t$. We set the diffusional dimensionality, $d = 2$, for the adsorbates moving on flat graphene and CNTs.

Once DA is adsorbed, the radial component of its MSD is negligible, and a 2D MSD can be calculated by considering its component in the directions axial to the CNT tube ($\text{MSD}_{\parallel}$) and its component in the azimuthal direction, which is around the CNT circumference and perpendicular to the CNT axis ($\text{MSD}_{\perp}$). To calculate these MSDs on the surface, we projected the adsorbate COM, $(r, \theta, z)$, onto an imaginary cylindrical surface which is coaxial with the CNT and has a radius equal to the ensemble average of the radial coordinate of the adsorbate, $(\langle r\rangle, \theta, z)$, where $\langle\cdot\rangle$ denotes the ensemble average, as illustrated in Fig. S1. The displacement along the $\perp$-direction is then $\langle r\rangle \cdot (\theta - \theta_0)$, and the displacement along the $\parallel$-direction is $z - z_0$, where $(\langle r\rangle, \theta_0, z_0)$ are the reference cylindrical COM coordinates at the beginning of each 10 ps trajectory segment. From there, the MSDs

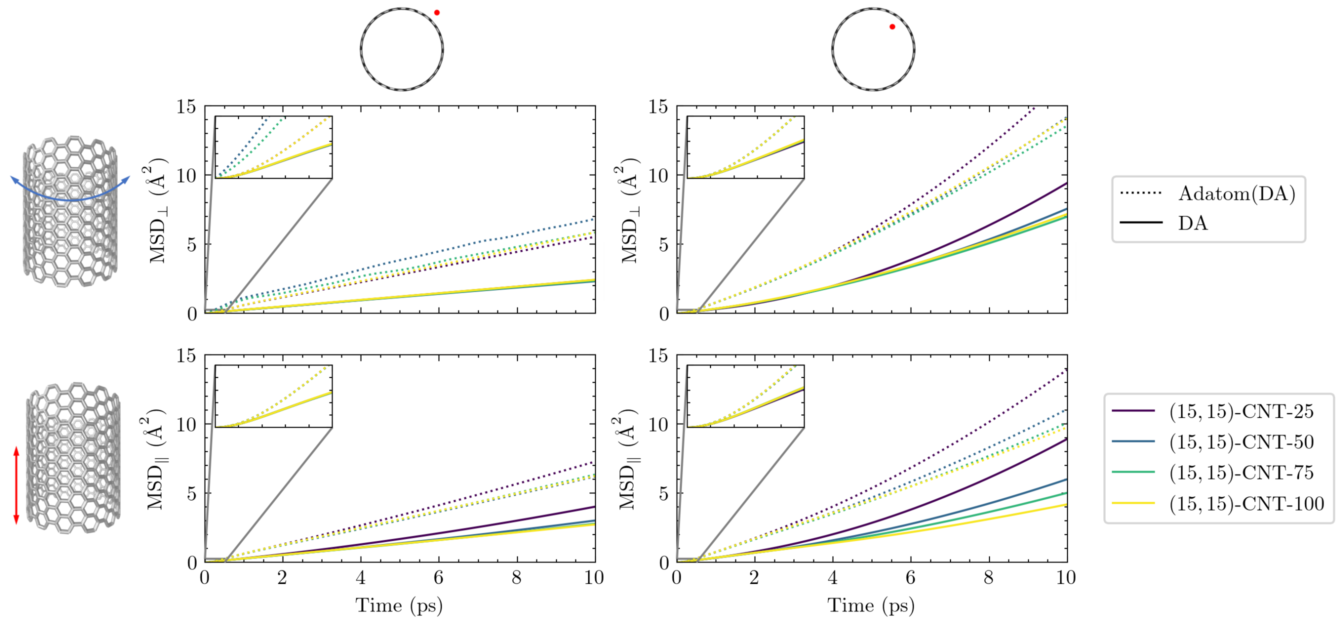


Figure S3: **The MSDs of Adatom(DA) and DA on** $(15,15)$**-CNTs of varying lengths.** The MSDs on the exterior and the interior of a $(15,15)$-CNT are shown in the left and right columns, respectively. The MSDs of adatom(DA) are shown in dashed curves, and the MSDs of DA are shown in solid curves. The top row shows the MSDs along the perpendicular ($\perp$) direction, while the bottom row shows the MSDs along the parallel ($\|$) direction. In the legend, $(15,15)$-CNT-25 to $(15,15)$-CNT-100, represent four armchair CNTs with lengths ranging from $25-100$Å (precise lengths are listed in Table S2).

were computed from the projected cylindrical coordinates of the adsorbate in the $\perp$- and $\|$-directions, according to:

$$\begin{aligned} \mathrm{MSD}_\perp &= \langle r\rangle^2 \cdot \langle(\theta-\theta_0)^2\rangle, \\ \mathrm{MSD}_\| &= \langle(z-z_0)^2\rangle, \\ \mathrm{MSD} &= \mathrm{MSD}_\perp + \mathrm{MSD}_\|. \end{aligned} \tag{S5}$$

**Additional Data Analysis Details.** All presented results were obtained from ten independent 5 ns trajectories at 300K with recording intervals of 0.01 ps. Diffusion coefficients were computed from linearly fit MSD curves using the Einstein relation, Eqn. S4, from 4-10 ps, and their standard deviations are reported across the MSD curves from the ten independent runs.

On the flat graphene surface, the $x$-direction is zigzag and the $y$-direction is armchair. On the armchair CNT ($(15,15)$-CNT), the $\|$-direction is zigzag and the $\perp$-direction is armchair; on the zigzag CNT ($(0,26)$-CNT), the $\perp$-direction is zigzag and the $\|$-direction is armchair. Therefore, in Figure 5, graphene $D_x$ is compared with $(15,15)$-CNT $D_\|$ and $(0,26)$-CNT $D_\perp$, and graphene $D_y$ is compared with $(15,15)$-CNT $D_\perp$ and $(0,26)$-CNT $D_\|$.

# 3 Finite Size Effects

Finite system size effects are a known issue when calculating diffusion constants within MD simulations, since the periodic boundary conditions needed to approximate the bulk system introduce unphysical hydrodynamic effects [37,38]. The dependence of this effect on simulation box size and shape is complex [46], and there is no precise correction that has been worked out for diffusion on the types of surfaces examined in this work. However, an approximate extrapolation to the infinite size system can be made using

$$D_{\mathrm{PBC}} = D_\infty - \frac{\alpha}{L}, \tag{S6}$$

where $D_{\mathrm{PBC}}$ is the diffusion coefficient computed from MD simulations in a finite simulation box under PBCs, $D_\infty$ is the diffusion coefficient for the infinite system, $L$ is the length of the cubic simulation box, and $\alpha$ is a system-specific constant that is independent of the sys-

Table S2: **Diffusion coefficients on $(15,15)$-CNTs of varying lengths.** The listed CNTs have identical diameters and helicities, however their lengths in the axial, $z$-direction, are varied from 25 Å to 100 Å. The sizes of the periodic cuboid simulation boxes are determined by the CNT lengths in the $z$ direction and extend for 15 Å in the $(x, y)$ dimensions away from the CNT. $D$ values reported here are the 2D diffusion coefficients fit from the MSD curves using the Einstein relation, Eqn. S4, from 4-10 ps.

| Sides | Surfaces | $z$ (Å) | Adatom(DA) | | DA | |
|---|---|---|---|---|---|---|
| | | | $D$ ($\times 10^{-5}$ cm$^2$/s) | $R^2$ | $D$ ($\times 10^{-5}$ cm$^2$/s) | $R^2$ |
| Exterior | $(15,15)$-CNT-25 | 24.56 | $3.29 \pm 0.18$ | 0.9999 | $1.74 \pm 0.10$ | 0.9992 |
| | $(15,15)$-CNT-50 | 49.12 | $3.08 \pm 0.48$ | 0.9998 | $1.38 \pm 0.11$ | 0.9997 |
| | $(15,15)$-CNT-75 | 73.68 | $2.97 \pm 0.15$ | 0.9998 | $1.31 \pm 0.05$ | 0.9999 |
| | $(15,15)$-CNT-100 | 100.698 | $3.03 \pm 0.15$ | 1.0 | $1.30 \pm 0.04$ | 0.9999 |
| Interior | $(15,15)$-CNT-25 | 24.56 | $9.35 \pm 0.83$ | 0.9958 | $5.97 \pm 0.63$ | 0.9923 |
| | $(15,15)$-CNT-50 | 49.12 | $7.22 \pm 0.43$ | 0.9980 | $4.19 \pm 0.34$ | 0.9948 |
| | $(15,15)$-CNT-75 | 73.68 | $6.62 \pm 0.50$ | 0.9988 | $3.61 \pm 0.22$ | 0.9966 |
| | $(15,15)$-CNT-100 | 100.698 | $6.61 \pm 0.52$ | 0.9989 | $3.34 \pm 0.26$ | 0.9973 |

tem size [39]. A more comprehensive discussion of this equation and its applicability to our system can be found in Ref. [18].

In our previous work [18], we found that a good fit to this equation could be obtained from simulations spanning a range of system sizes, enabling the extrapolation of $D_\infty$ values. We follow the same approach here for adatom(DA) and DA on both the interiors and exteriors of $(15,15)$-CNTs of varying lengths, ranging from 25-100 Å. The resulting MSD plots are shown in Figure S3, and the diffusion constants, $D$, from these differently-sized simulations are shown in Table S2.

Figure S4 displays a series of plots from the data in Table 1, where the diffusion constants, $D$, from the finite systems are plotted against the inverse length of the CNT, $L^{-1}$, and a linear fit is obtained to enable the extrapolation of the diffusion constants on an infinite CNT surface, $D_\infty$. As expected, we find that the system size dependence is stronger for an adsorbate on the CNT's interior.

The standard errors of $D_\infty$ in Table 1 and Figure 13a were computed using

$$s_{\hat{\alpha}} = \sqrt{\frac{1}{n \times (n-2)} \times \frac{\sum^{n}(y_i - \hat{y}_i)^2}{\sum^{n}(x_i - \hat{x}_i)^2}}, \quad \text{(S7)}$$

where the $s_{\hat{\alpha}}$ is the standard error of the estimated intercept ($\hat{\alpha}$), $n$ is the number of observations. For the results listed in Table 1, $n = 40$ for the CNT observations and $n = 50$ for the graphene observations. $(x_i, y_i)$ and $(\hat{x}_i, \hat{y}_i)$ are the $i^{th}$ observed and regressed $(L^{-1}, D)$ data pairs, respectively. This standard error approach assumed the residuals are normally distributed.

# 4 Additional Results.

Figure S5 displays the number density of water next to the different CNT and graphene surfaces. This plot was also used as the basis of the choice of 5 Å as the cutoff value when assessing the number of waters in the first solvation shell around DA in Fig. 11.

Figure S6 displays the tilt angle, $\phi$, and the alignment with the CNT axis, measured by $\theta$, for DOQ at different carbon:vacuum interfaces, showing that the trends observed for DA in Fig. 9d and Fig. 10d also hold for DOQ.

Finally, Figure S7 shows the frequency of jumps from one side of the CNT groove to the other for several trajectories of DA at both the solvated (Fig. S7a) and vacuum (Fig. S7b) interfaces.

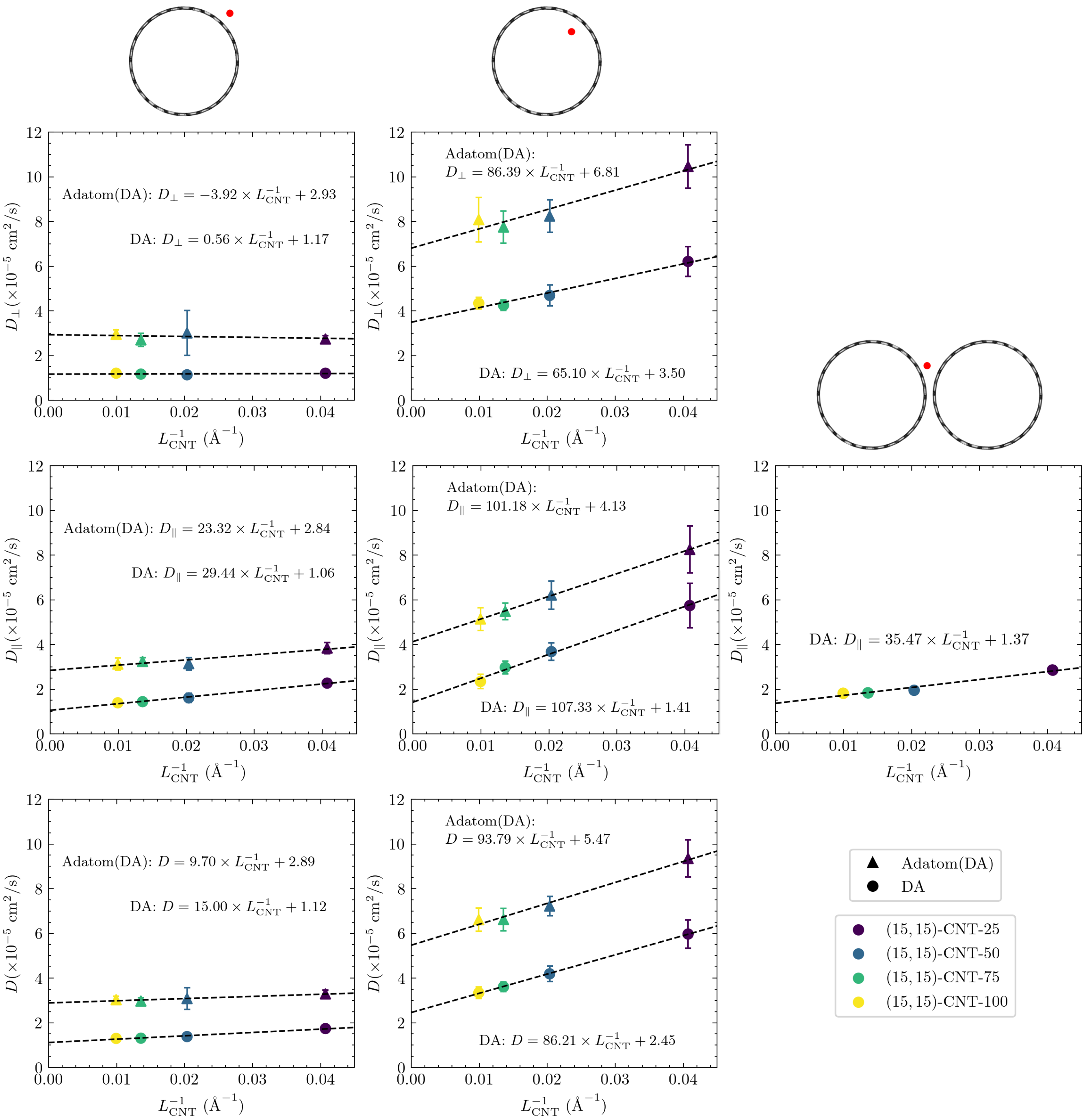


Figure S4: **Diffusion coefficients of Adatom(DA) and DA on CNTs and CNT grooves of varying lengths.** The diffusion coefficients on the exterior (left column) and interior (middle column) of the (15, 15)-CNT, and on the CNT groove (right column), are plotted against the inverse length of the simulation box, characterized by the length of the CNT, ($L_{\mathrm{CNT}}$). The dashed lines are linear fits to the $D$ values calculated from simulations of different sizes. The y-intercepts indicate the extrapolated diffusion coefficients on an infinite surface, $D_\infty$.

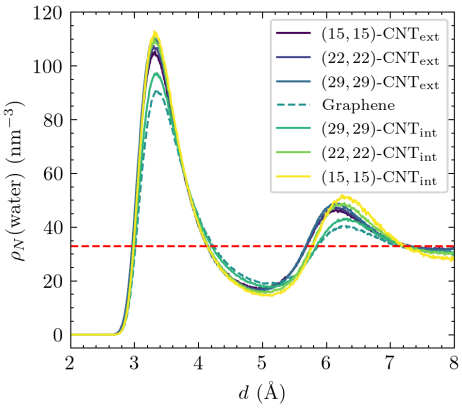


Figure S5: **Number density of water near differently-curved carbon surfaces.** The number density of water, $\rho_N$(water), is computed vs. the distance from the carbon surface using the location of the oxygen atoms. The horizontal red dashed line marks the reference density of 33 nm$^{-3}$, which is the number density of bulk water at 300K. The results are collected from ten 5 ns $NVT$ trajectories at 300K at a recording interval of 5 ps.

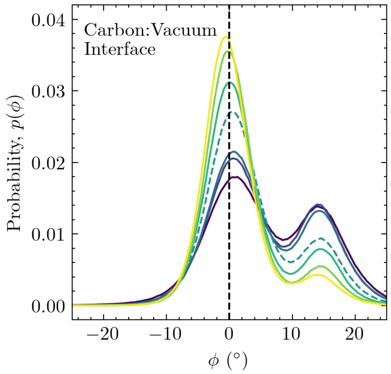


][]
(a)

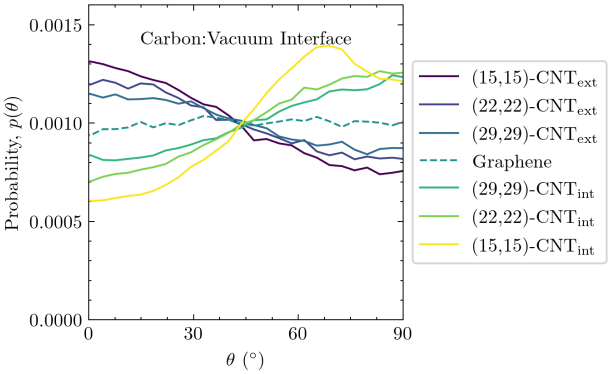


][]
(b)

Figure S6: **Tilt and orientational angle distributions of DOQ at the carbon:vacuum interface.** Distributions for (a) the tilt angle, $\phi$, and (b) the orientational axial alignment angle, $\theta$, of DOQ at the carbon:vacuum interface are plotted for differently-curved surfaces.

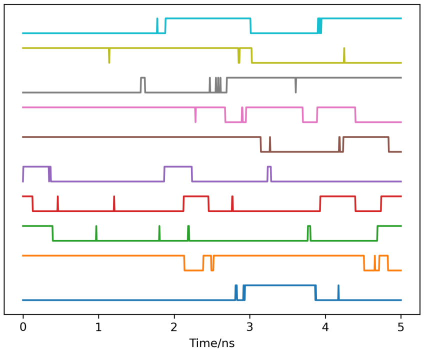


][]
(a)

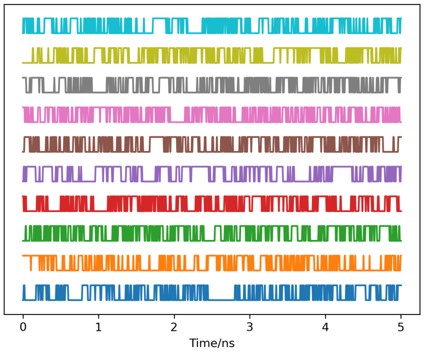


][]
(b)

Figure S7: **Trajectories of DA jumping across the CNT groove.** Each of the ten differently colored lines indicates the jumps observed from one CNT surface to the other within a single trajectory. In all cases, DA remains in the groove after the jump. The high and low levels in these trajectories represent DA on the two different sides of the groove – its location was determined by calculating which CNT axis was closer to the center of the aromatic ring. Results are shown (a) for the solvated groove and (b) for the groove in a vacuum. The sampling frequency is 5 ps.
